# Supplementary material for: In Silico and Chromatographic Methods for Analysis of Biotransformation of Prospective Neuroprotective Pyrrole-Based Hydrazone in Isolated Rat Hepatocytes
Source: Molecules. 2024 Mar 26;29(7):1474. doi: 10.3390/molecules29071474 (PMC11013089; doi:10.3390/molecules29071474)

In Silico and Chromatographic Methods for Analysis of Biotransformation of Prospective Neuroprotective Pyrrole-Based Hydrazone in Isolated Rat Hepatocytes

Supplementary Materials

Table S1. BioTrasformer 3.0 data for the title pyrrole.

№	Metabolite	Results
M1_11b	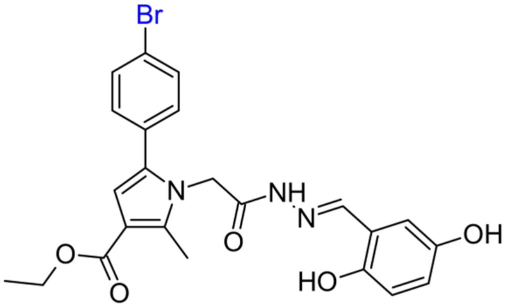	Isotopic mass: 499.07<br>CYP-isoform: 1A2<br>Metabolic reaction:<br>Aromatic hydroxylation
M2_11b	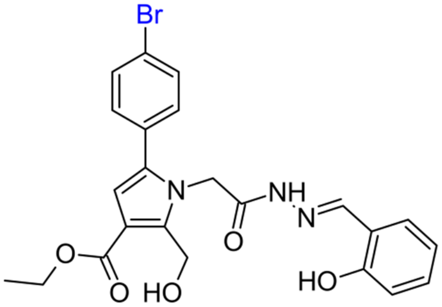	Isotopic mass: 499.07<br>CYP-isoform: 1A2<br>Metabolic reaction:<br>Aliphatic hydroxylation
M3_11b	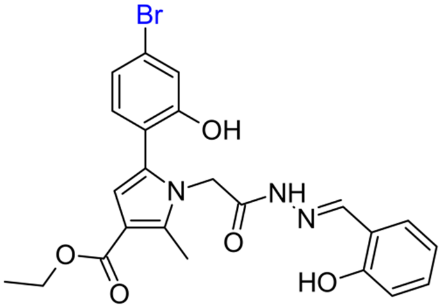	Isotopic mass: 499.07<br>CYP-isoform: 1A2<br>Metabolic reaction:<br>Aromatic hydroxylation
M4_11b	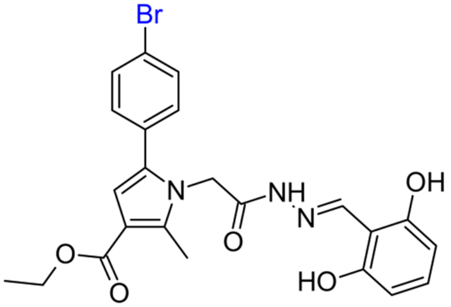	Isotopic mass: 499.07<br>CYP-isoform: 1A2<br>Metabolic reaction:<br>Aromatic hydroxylation

M5_11b	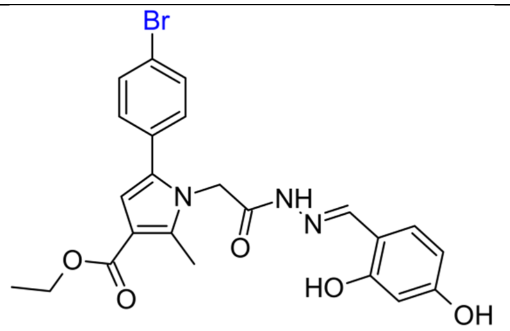	Isotopic mass: 499.07<br>CYP-isoform: 1A2<br>Metabolic reaction:<br>Aromatic hydroxylation
M6_11b	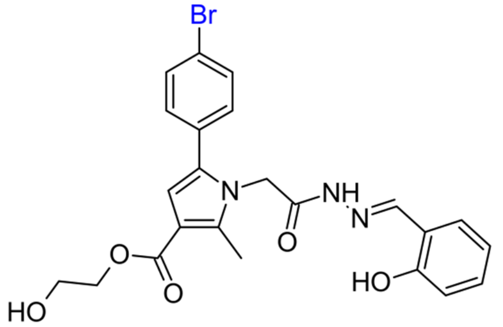	Isotopic mass: 499.07<br>CYP-isoform: 1A2<br>Metabolic reaction:<br>Aliphatic hydroxylation
M7_11b	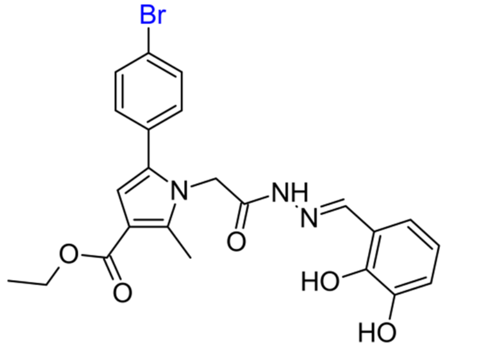	Isotopic mass: 499.07<br>CYP-isoform: 1A2<br>Metabolic reaction:<br>Aromatic hydroxylation
M8_11b	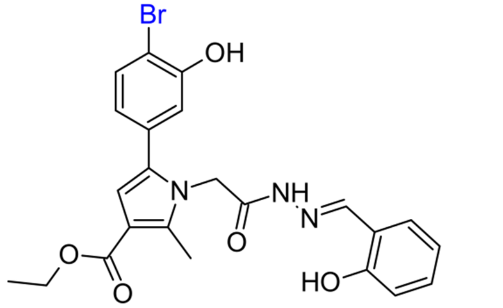	Isotopic mass: 499.07<br>CYP-isoform: 1A2<br>Metabolic reaction:<br>Aromatic hydroxylation
M9_11b	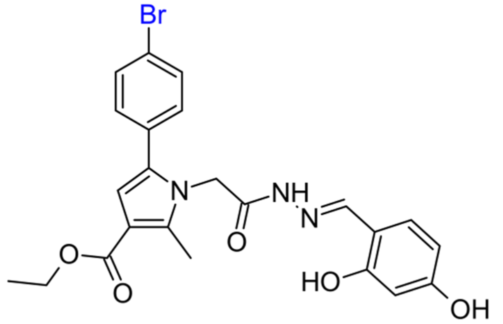	Isotopic mass: 499.07<br>CYP-isoform: 2C9<br>Metabolic reaction:<br>Aromatic hydroxylation

M10_11b	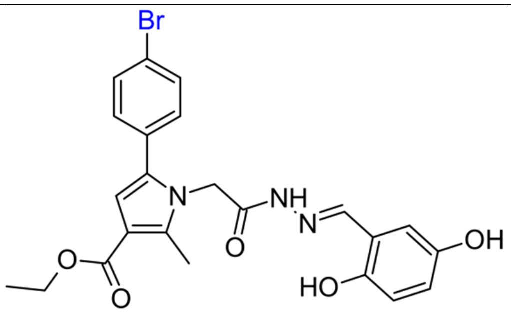	Isotopic mass: 499.07<br>CYP-isoform: 3A4<br>Metabolic reaction:<br>Aromatic hydroxylation
M11_11b	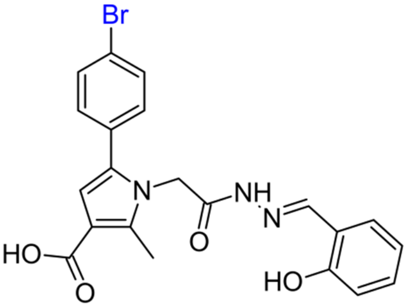	Isotopic mass: 455.04<br>CYP-isoform: 2C8<br>Metabolic reaction:<br>O-dealkylation
M12_11b	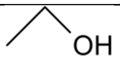	Isotopic mass:46.04<br>CYP-isoform: 2C8<br>Metabolic reaction:<br>O-dealkylation

Table S2. BioTransformer 3.0 data for phase II of metabolism.

№	**Metabolite**	**Results**
1.	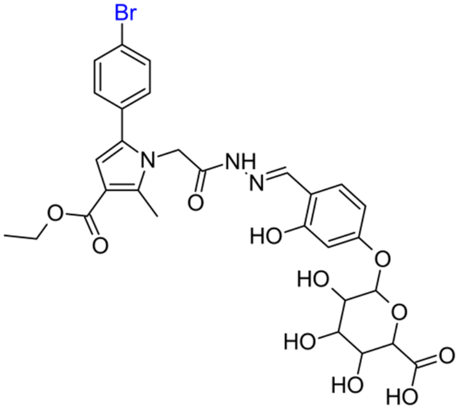	Isotopic mass: 675.11<br>Enzyme: UDP-glucuronosyltransferase<br>Metabolic reaction:<br>Glucuronidation

2.	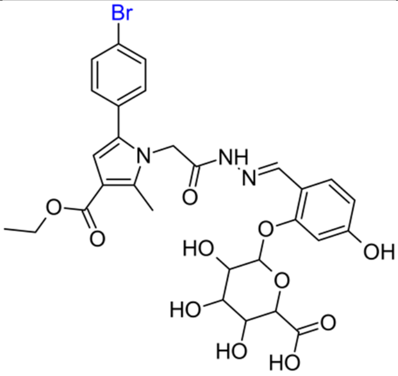	Isotopic mass: 675.11<br>Enzyme: UDP-glucuronosyltransferase<br>Metabolic reaction: Glucuronidation
3.	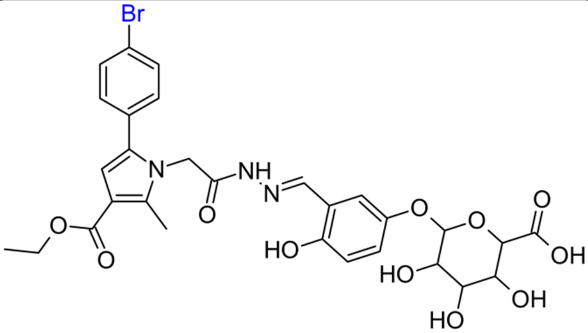	Isotopic mass: 675.11<br>Enzyme: UDP-glucuronosyltransferase<br>Metabolic reaction: Glucuronidation
4.	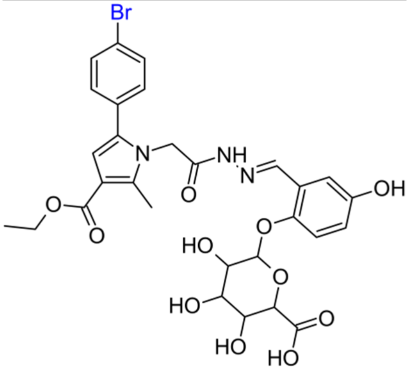	Isotopic mass: 675.11<br>Enzyme: UDP-glucuronosyltransferase<br>Metabolic reaction: Glucuronidation
5.	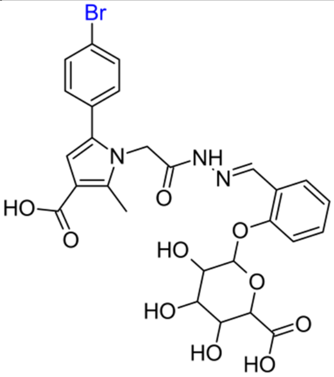	Isotopic mass: 631.08<br>Enzyme: UDP-glucuronosyltransferase<br>Metabolic reaction: Glucuronidation

6.	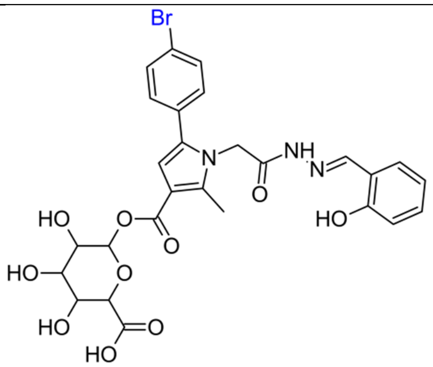	Isotopic mass: 631.08<br>Enzyme: UDP-glucuronosyltransferase<br>Metabolic reaction: Glucuronidation
7.	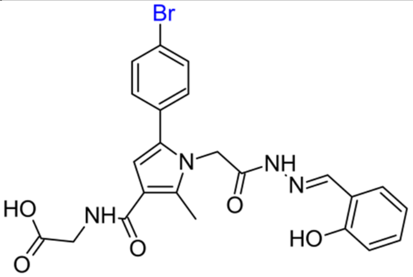	Isotopic mass: 512.07<br>Enzyme: Glycine- N-acetyltransferase<br>Metabolic reaction: Glycine conjugation

Figure S1. MS2 spectrum of compounds M9_11b and M10_11b

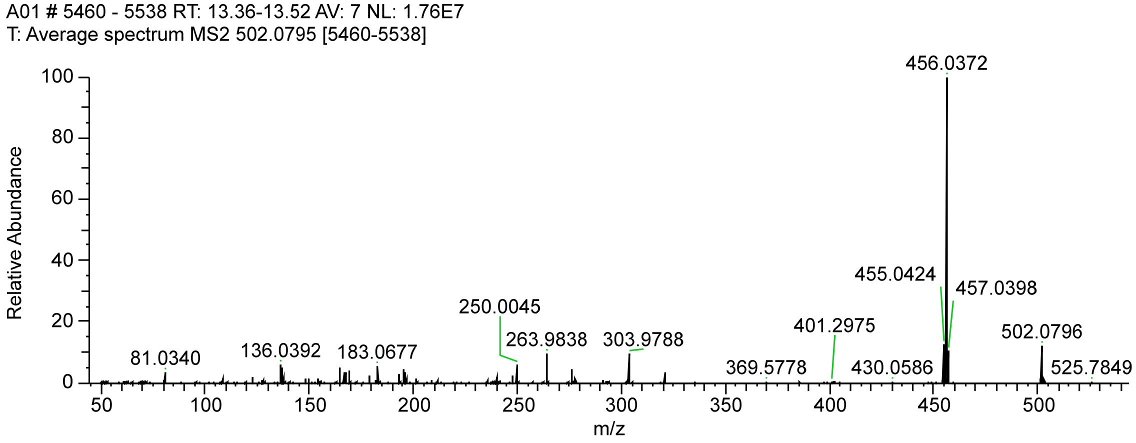

A03 #5470 RT: 13.27 AV: 1 NL: 3.63E7
T: FTMS + p ESI d Full ms2 502.0791@hcd33.33 [50.0000-530.0000]

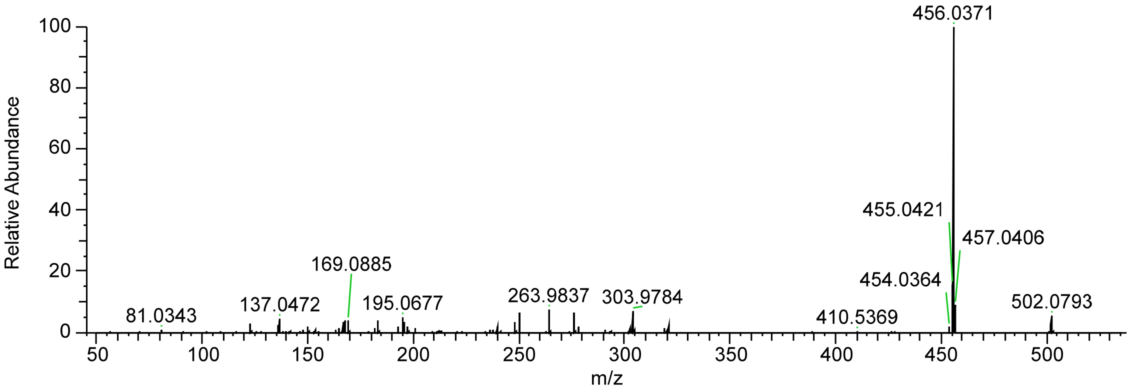

Supplement: Supplementary file 1 [file molecules-29-01474-s001.zip › molecules-2890557-supplementary.pdf]
